# Galectin-3 Inhibitors Suppress Anoikis Resistance and Invasive Capacity in Thyroid Cancer Cells

**DOI:** 10.1155/2021/5583491

**Published:** 2021-05-07

**Authors:** Jie-Jen Lee, Yi-Chiung Hsu, Ying-Syuan Li, Shih-Ping Cheng

**Affiliations:** ^1^Department of Surgery, MacKay Memorial Hospital and Mackay Medical College, Taipei 104215, Taiwan; ^2^Department of Biomedical Sciences and Engineering, National Central University, Taoyuan City 320317, Taiwan; ^3^Department of Medical Research, MacKay Memorial Hospital, Taipei 104215, Taiwan; ^4^Department of Pharmacology, School of Medicine, College of Medicine, Taipei Medical University, Taipei 110301, Taiwan

## Abstract

Accumulating evidence suggests that galectin-3 is a histologic marker of thyroid cancer. However, the pharmacological lectin-based approach has not been well studied. In the present study, we aimed to investigate the therapeutic potential of novel galectin-3 inhibitors by treating thyroid cancer cells with different concentrations of GB1107 or TD139. At high doses, TD139, but not GB1107, reduced cell viability and clonogenicity of thyroid cancer cells. TD139 induced apoptosis of thyroid cancer cells, as evident by an increase in the percentage of sub-G1 cells on cell cycle analysis, caspase-3 activation, and PARP1 cleavage. Either GB1107 or TD139 significantly inhibited cell coherence and counteracted anoikis resistance. Both inhibitors decreased migratory and invasive abilities in a dose-dependent manner. Furthermore, GB1107 and TD139 treatment attenuated AKT phosphorylation and decreased the expression of *β*-catenin and MMP2. In conclusion, these novel galectin-3 inhibitors suppressed the anoikis resistance, motility, and invasive capacity of thyroid cancer cells at least partly through the AKT/*β*-catenin pathway. Galectin-3 inhibitors are potentially suitable for preclinical evaluation of treatment and/or prevention of metastatic spread in thyroid cancer.

## 1. Introduction

Thyroid nodules are a common finding, particularly in women and in the elderly, while thyroid carcinoma is relatively uncommon [1]. Other than clinical assessment, thyroid ultrasonography and fine-needle aspiration biopsy are the primary tools used for initial cancer risk stratification. Nonetheless, about 20% to 25% of cytological evaluation would be reported as indeterminate. A few molecular testing modalities have been developed to minimize unnecessary diagnostic thyroid surgery for indeterminate thyroid nodules [2]. There has been considerable interest in the identification of cancer-specific markers by gene expression profiles or immunocytochemistry. Among various biomarkers, galectin-3 and dipeptidyl peptidase IV (DPP4) were found to be upregulated and consistently coexpressed in differentiated thyroid cancer [3]. Recently, we reported that DPP4 is not only a diagnostic and prognostic marker but also a potential therapeutic target in papillary thyroid cancer [4].

Galectins belong to the lectin superfamily and exert biological functions by interacting with glycoconjugates. Altered expression or function in galectins has frequently been associated with cancer [5]. Accumulating evidence points towards high reliability of the utilization of galectin-3 for thyroid cancer diagnosis at histology [6]. However, strategies to target galectin-3 for thyroid cancer therapy are less well studied. Menachem et al. reported that combined treatment with a Ras inhibitor and modified citrus pectin, a water-soluble polysaccharide derived from a citrus fruit that inhibits galectin-3, induced cell cycle arrest and apoptosis of thyroid cancer cells [7]. With a deeper understanding of the crystallographic structure of galectins, more potent inhibitors with high affinity have been developed [8]. In the present study, we aimed to explore the therapeutic potential of novel galectin-3 inhibitors in thyroid cancer.

## 2. Materials and Methods

### 2.1. Cell Culture and Reagents

Human thyroid cancer cell lines FTC-133 and 8505C were purchased from the European Collection of Authenticated Cell Cultures (ECACC, Salisbury, UK). The human thyroid follicular epithelial cell line Nthy-ori 3-1 (abbreviated as Nthy thereafter) was also obtained from the ECACC. Cell line authentication has been periodically monitored by cell morphology and short tandem repeat profiling. Mycoplasma testing in cell cultures was regularly performed. Galectin-3 inhibitor GB1107 was purchased from MedChemExpress, Monmouth Junction, NJ, USA, and TD139 from BioVision, Milpitas, CA, USA.

### 2.2. Cell Viability Assay

FTC-133, 8505C, and Nthy cells were seeded in 96-well plates at 1 × 10^4^ cells/well and cultured overnight. Subsequently, cells were treated with increasing concentrations of GB1107 or TD139 (0, 10, and 100 *μ*M) for 24 to 72 h. Dimethyl sulfoxide (DMSO) served as vehicle control. Cell viability was determined using the Cell Counting Kit-8 (CCK-8; Sigma-Aldrich, St. Louis, MO, USA) [9]. The absorbance at 450 nm was measured with Varioskan Flash Microplate Reader (Thermo Fisher Scientific, Vantaa, Finland).

### 2.3. Cell Cycle Analysis

Cells were cultured in six-well plates (4 × 10^5^ cells/well), synchronized by serum starvation, and stimulated to enter the cell cycle by the addition of fresh complete medium containing different concentrations of GB1107 or TD139 (0, 10, and 100 *μ*M) for 24 h. Cells were then harvested and fixed with 70% ethanol at 4°C overnight. Subsequently, fixed cells were washed and incubated with propidium iodide (PI; 5 *μ*g/ml; Sigma-Aldrich). Data acquisition was carried out using a FACSCalibur flow cytometer (BD Biosciences, San Jose, CA, USA) and analyzed with CellQuest Pro software, as we previously reported [10].

### 2.4. Clonogenic Assay

For the colony-forming assay, FTC-133 and 8505C cells were seeded in six-well plates at 500 cells per well in the presence of increasing concentrations of GB1107 or TD139 (0, 10, and 100 *μ*M). After ten days, cells were stained with 3% crystal violet. The number of formed colonies was counted under the microscope [11].

### 2.5. Anoikis Assay

Anoikis assay was performed as described previously [12]. Briefly, poly(2-hydroxyethyl methacrylate) (poly-HEMA; Sigma-Aldrich) was dissolved in 95% ethanol to prepare a 20 mg/ml solution. Sterile six-well plates were coated with 700 *μ*l/well poly-HEMA. Subsequently, cells in different concentrations of GB1107 or TD139 were transferred into poly-HEMA-coated plates. After 48 h, cells were stained with Hoechst 33342 (Abcam, Cambridge, UK) and PI (Abcam), and cell morphology under the anchorage-independent condition was observed using a fluorescence microscope.

### 2.6. Migration and Invasion Assays

Transwell chambers with 8-*μ*m pore-size polycarbonate membrane (Corning Life Sciences, Tewksbury, MA, USA) were used. For migration assay, FTC-133 cells (0.8 × 10^4^ cells/well) or 8505C cells (1.5 × 10^4^ cells/well) resuspended in serum-free medium with different concentrations of GB1107 or TD139 were seeded onto the upper chamber, while the complete medium containing 10% fetal bovine serum as the chemoattractant was added to the lower chamber [13]. After 24 h, the migrated cells on the lower surface of the porous membrane were fixed and stained with Diff-Quik (Sysmex, Kobe, Japan). For invasion assay, FTC-133 cells (1 × 10^4^ cells/well), 8505C cells (1 × 10^4^ cells/well), or Nthy cells (2 × 10^4^ cells/well) were allowed to invade through BioCoat cell culture inserts precoated with Matrigel. The invaded cells were fixed and stained after 24 h. Migrated or invaded cells were photographed under the microscope, and five random fields were counted for each assay.

### 2.7. Immunoblotting

Total cellular protein was extracted from FTC-133 or 8505C cells treated with increasing concentrations of GB1107 or TD139 (0, 10, and 100 *μ*M) for 24 h. Equal amounts of protein were electrophoresed in 10% polyacrylamide gels and transferred to the nitrocellulose membrane. The membrane was blocked in 5% skim milk, incubated with the primary antibody at 4°C overnight, and then treated with secondary antibody at 37°C for 1 h [14]. The primary antibodies used for western blotting were purchased from Cell Signaling Technology, Danvers, MA, USA, unless otherwise specified: galectin-3 (1 : 1000 dilution; MABT51; Sigma-Aldrich), cyclin D1 (1 : 700; #2978), cleaved caspase-3 (1 : 700; #9661), PARP1 (1 : 700; ab32138; Abcam), cleaved PARP1 (1 : 700; ab32064; Abcam), phospho-p44/42 MAPK (ERK1/2) Thr202/Tyr204 (1 : 700; #9101), ERK1/2 (1 : 700; #9102), phospho-p38 MAPK Thr180/Tyr182 (1 : 700; #9216), p38 (1 : 700; #9212), phospho-JNK1 Thr183/Tyr185 (1 : 700; ab215208; Abcam), JNK1 (1 : 700; ab110724; Abcam), phospho-AKT Ser473 (1 : 700; #9271), AKT (1 : 700; #4691), *β*-catenin (1 : 700; #9562), MMP2 (1 : 500; MAB3308; Sigma-Aldrich), and *β*-actin (1 : 5000; A5441; Sigma-Aldrich).

### 2.8. Statistical Analysis

All experiments were carried out at least in triplicates. Data are presented as the mean ± standard deviation. Statistical analysis was performed using one-way analysis of variance (ANOVA), followed by Dunnett's post hoc analysis. A *p*-value of less than 0.05 was considered statistically significant.

## 3. Results

### 3.1. Effects of Galectin-3 Inhibitors on Cell Viability

**Figure 1:**
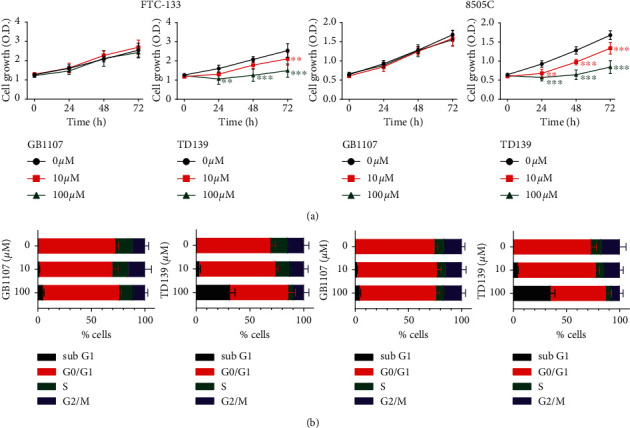
Effects of galectin-3 inhibitors (GB1107 and TD139) on cell growth and cell cycle distribution in FTC-133 and 8505C human thyroid cancer cells. (a) Cells were treated with GB1107 or TD139 of the indicated concentrations for 24 to 72 hours, and cell viability was determined using Cell Counting Kit-8 assay. O D., optical density value at 450 nm. (b) The distribution of cell cycle following treatment with GB1107 or TD139 for 24 h was determined using flow cytometry. ^*∗∗*^*p* < 0.01, ^*∗∗∗*^*p* < 0.001.

FTC-133 and 8505C cells were treated with increasing concentrations of two different galectin-3 inhibitors, GB1107 and TD139, and cell viability was assessed using CCK-8 assay. As shown in Figure 1(a), GB1107 did not influence cell viability in thyroid cancer cells. Nonetheless, TD139 reduced cell viability in a dose-dependent manner. Treatment with TD139 100 *μ*M for 72 h led to a reduction in cell viability of 41.1 ± 13.7% and 49.7 ± 10.3% in FTC-133 and 8505C cells, respectively.

Following the findings above, we further determined the alterations in cell cycle distribution associated with treatment with galectin-3 inhibitors. As expected, GB1107 did not change the distribution of cell cycle phases (Figure 1(b)). Nonetheless, the percentage of sub-G1 cells significantly increased to 31.3 ± 4.8% and 35.0 ± 4.1% in FTC-133 and 8505C cells, respectively, following treatment with TD139 100 *μ*M for 24 h.

**Figure 2:**
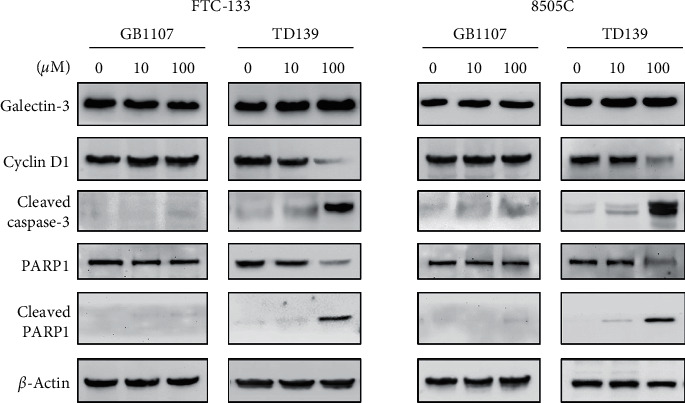
Expression of galectin-3, cyclin D1, and apoptosis-relevant proteins following treatment with galectin-3 inhibitors (GB1107 and TD139) in FTC-133 and 8505C thyroid cancer cells. The protein expression was determined using immunoblotting after 24-h treatment.

We further examined whether galectin-3 inhibitors influence the expression of galectin-3. As shown in Figure 2, GB1107 and TD139 did not change the expression of galectin-3 in thyroid cancer cells. The expression of cyclin D1 was attenuated by treatment with a high dose (100 *μ*M) of TD139, consistent with a decrease in the percentage of S-phase cells on cell cycle analysis. Of note, TD139 at 100 *μ*M significantly increased the cleaved active caspase-3. During the process of apoptosis, caspases-3 activation results in cleavage and inactivation of proteins involved in DNA repair. Among these proteins, poly(ADP-ribose) polymerase-1 (PARP1) cleavage is a frequently used marker of apoptosis. We observed that remarkable PARP1 cleavage was present at 24 h following treatment with TD139 (Figure 2), in agreement with the sub-G1 population representing apoptotic cells with reduced DNA content (Figure 1(b)). Taken together, these results indicate that TD139, but not GB1107, induces apoptosis and reduces cell viability in thyroid cancer cells.

### 3.2. Effects of Galectin-3 Inhibitors on Clonogenicity and Anoikis Resistance

**Figure 3:**
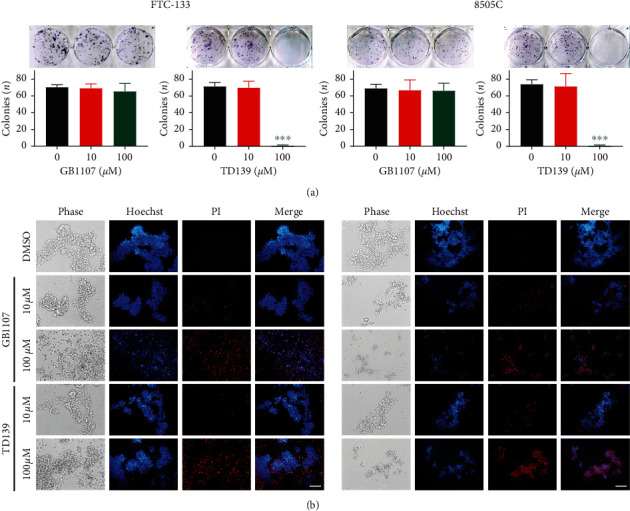
Effects of galectin-3 inhibitors (GB1107 and TD139) on clonogenicity and anoikis resistance in FTC-133 and 8505C human thyroid cancer cells. (a) Cells treated with GB1107 or TD139 were subjected to clonogenic assay. (b) Cells grown on poly-HEMA-coated plates were treated with GB1107 or TD139 for 48 h and stained with Hoechst 33342 and propidium iodide (PI). Scale bars, 100 *μ*m. ^*∗∗∗*^*p* < 0.001.

To further explore the impact of galectin-3 inhibitors on the colony-forming capability of thyroid cancer cells, FTC-133 and 8505C cells were treated with GB1107 or TD139 for 10 days. As shown in Figure 3(a), GB1107 did not influence the colony-forming efficiency in thyroid cancer cells. On the contrary, treatment with TD139 100 *μ*M significantly abrogated the colony-forming ability of FTC-133 and 8505C cells.

Normal cells undergo a programmed cell death known as anoikis while losing contact with the extracellular matrix or neighboring cells. During cancer progression and metastasis, cancer cells may develop anoikis resistance and sustain adherence-independent cell growth [15]. We next examined the effects of GB1107 and TD139 on anoikis induced by culturing cells in suspension using poly-HEMA-coated plates. Interestingly, treatment with either GB1107 or TD139 at a concentration of 100 *μ*M significantly inhibited cell coherence and induced remarkable cell death, as evident by an increase in PI‐staining positive cells (Figure 3(b)). These findings suggest that inhibition of anoikis resistance is a more universal property of galectin-3 inhibitors.

### 3.3. Effects of Galectin-3 Inhibitors on Cell Migration and Invasion

**Figure 4:**
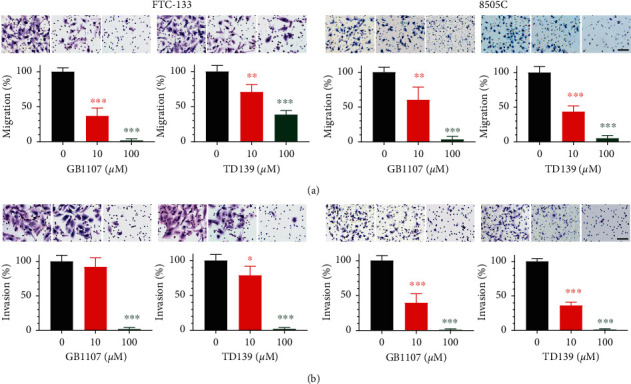
Effects of galectin-3 inhibitors (GB1107 and TD139) on cell migration and invasion in FTC-133 and 8505C human thyroid cancer cells. (a) Cells were allowed to migrate through Transwell chambers in the presence of GB1107 or TD139 for 24 h. (b) Cells invading through Matrigel-coated Transwell chambers with or without 24 h GB1107 and TD139 treatment were fixed and quantified. Scale bars, 100 *μ*m. ^*∗*^*p* < 0.05, ^*∗∗*^*p* < 0.01, ^*∗∗∗*^*p* < 0.001.

The migration and invasion abilities of thyroid cancer cells following treatment with galectin-3 inhibitors were further determined. We found that GB1107 and TD139 consistently inhibited the migratory and invasive behavior of both thyroid cancer cell lines in a dose-dependent fashion (Figure 4). Collectively, these results indicate that galectin-3 inhibitors mainly affect anchorage-independent growth and motility of thyroid cancer cells.

### 3.4. Effects of Galectin-3 Inhibitors on a Follicular Epithelial Cell Line

**Figure 5:**
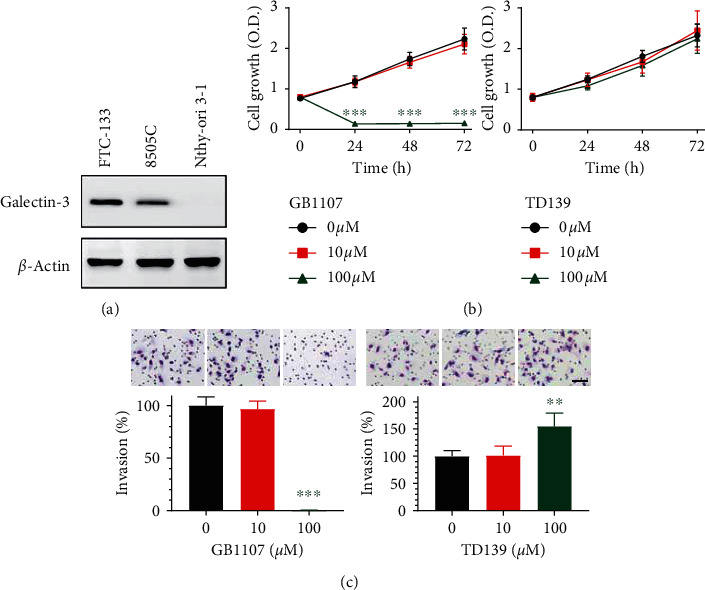
Effects of galectin-3 inhibitors (GB1107 and TD139) on cell growth and invasion in the human thyroid follicular epithelial cell line Nthy-ori 3-1. (a) Protein expression of galectin-3 in thyroid cancer cell lines (FTC-133 and 8505C) and Nthy-ori 3-1. (b) Nthy-ori 3-1 cells were treated with GB1107 or TD139 of the indicated concentrations for 24 to 72 h and cell viability was determined using Cell Counting Kit-8 assay. OD: optical density value at 450 nm. (c) Nthy-ori 3-1 cells invading through Matrigel-coated Transwell chambers with or without 24-h GB1107 and TD139 treatment were fixed and quantified. Scale bars, 100 *μ*m. ^*∗∗*^*p* < 0.01, ^*∗∗∗*^*p* < 0.001.

The immortalized human thyroid follicular epithelial cell line Nthy-ori 3-1 was used to evaluate the effects of galectin-3 inhibitors on non-transformed cells. As expected, the expression of galectin-3 was hardly detectable in Nthy cells (Figure 5(a)). We found that Nthy cells were vulnerable to GB1107 at 100 *μ*M (Figure 5(b)). Otherwise, the cell viability of Nthy cells was not affected by treatment with TD139. Invaded Nthy cells were barely observed following treatment with GB1107 at 100 *μ*M, probably due to the induced cytotoxicity (Figure 5(c)). Paradoxically, TD139 treatment stimulated invasion of Nthy cells at a high dose (100 *μ*M). Collectively, the responses to galectin-3 inhibitors were different in nonmalignant follicular epithelial cells compared to thyroid cancer cells, and the difference may result from differential expression of galectin-3.

### 3.5. Downstream Molecular Effectors of Galectin-3 Inhibitors in Thyroid Cancer Cells

**Figure 6:**
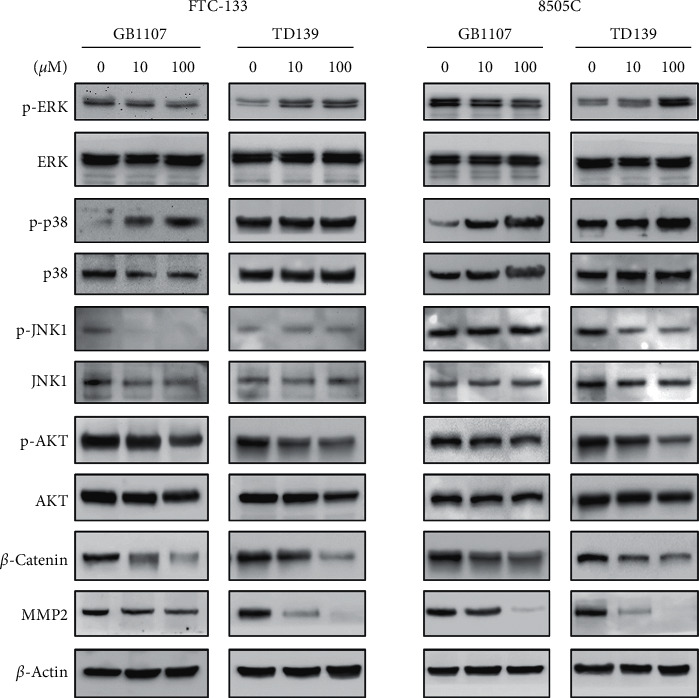
Expression of relevant signaling molecules following treatment with galectin-3 inhibitors (GB1107 and TD139) in FTC-133 and 8505C human thyroid cancer cells. The protein expression was determined using immunoblotting after 24-h treatment.

To further investigate the potential mechanism involved in the effects of galectin-3 inhibitors, we evaluated the protein expression of relevant signaling molecules (Figure 6). In the two cell lines we examined, GB1107 treatment decreased ERK phosphorylation, whereas TD139 treatment increased ERK phosphorylation. On the other hand, phosphorylation of p38 MAPK increased following treatment with GB1107, but not TD139. JNK1 phosphorylation tended to decrease following treatment with galectin-3 inhibitors, but the pattern was not consistent between the two cell lines (Figure 6). Overall, these findings suggest that the mitogen-activated protein kinase (MAPK) pathways may not be the main effectors of the galectin-3 inhibitors. By contrast, both GB1107 and TD139 attenuated AKT phosphorylation in FTC-133 and 8505C cells. Furthermore, the expression of *β*-catenin showed a corresponding decrease following GB1107 or TD139 treatment. Finally, we examined the expression of matrix metalloproteinase MMP2 in thyroid cancer cells. Parallel to the decreased invasive capacity in association with treatment with galectin-3 inhibitors, MMP2 expression progressively diminished when thyroid cancer cells were treated with increasing doses of GB1107 or TD139. Taken together, the AKT/*β*-catenin pathway is likely involved in the suppression of anoikis resistance and motility in thyroid cancer cells following treatment with galectin-3 inhibitors.

## 4. Discussion

Galectin-3 is a versatile carbohydrate-binding protein with a high affinity for *β*-galactosides and regulates pleiotropic biological functions such as cell adhesion and cell-cell interactions [16]. The development of galectin-3 inhibitors has been an intense area of interest for years as galectin-3 targeting may be exploited in diverse physiological and pathological conditions. Galectin-3-knockout mice are viable and have no overt abnormalities [17]. Moreover, galectin inhibitors generally have low toxicity profiles [18]. Given that galectin-3 shares structural similarities with the spike proteins of the *β*-genus of coronaviridae, including SARS-CoV-2, galectin-3 inhibitors are of particular importance in the era of COVID-19 [19].

Galectin-3 has a good discriminative power to differentiate benign thyroid nodules from cancer. Some studies reported that serum galectin-3 levels were higher in thyroid cancer patients, although inconsistencies exist across studies [20]. Additionally, radiolabeled antibodies or antigen-binding fragments against galectin-3 have been evaluated as molecular imaging probes in animal models [21]. Therefore, it is intriguing to assess the therapeutic potential of targeting galectin-3 in thyroid cancer. Lin and colleagues first demonstrated that a small molecule inhibitor of galectin-3 (Td131_1) promoted apoptosis, enhanced chemosensitivity to doxorubicin, and improved radiosensitivity in thyroid cancer cells [22]. Subsequently, modified citrus pectin was shown to induce cell cycle arrest and apoptosis when combined with a Ras inhibitor [7]. With the advent of pharmaceutical agents, it is worth investigating the roles of novel galectin-3 inhibitors.

In the present study, we examined the effects of GB1107 and TD139 in thyroid cancer cells. GB1107 is a newly developed, selective monosaccharide galectin-3 inhibitor with low clearance and good uptake upon oral administration [23]. Recently, GB1107 was shown to reduce lung adenocarcinoma growth, block metastasis, and potentiate the effects of a PD-L1 immune checkpoint inhibitor [24]. TD139 is the most potent inhibitor of thio-digalactosides derivatives and has been used in clinical trials for the treatment of patients with idiopathic pulmonary fibrosis (IPF) [25]. Recent studies have confirmed that TD139 is safe and well tolerated in healthy subjects and IPF patients [26]. There is another galectin-3 inhibitor, belapectin (GR-MD-02), that has been tested in clinical trials but was not included in this study. In a multicenter phase 2b study, belapectin was safe but did not significantly improve fibrosis or hepatic venous pressure gradient among patients with nonalcoholic steatohepatitis with cirrhosis and portal hypertension [27].

Although previous studies suggest that galectin-3 inhibition led to apoptosis in thyroid cancer cells [22, 28], we found that this effect was limited to TD139 but not GB1107. The reason behind this discrepancy remains unclear. Knockdown of galectin-3 decreased cell growth in B-CPAP but not 8305C cells [29], suggesting cell-dependent disparities. The most consistent finding in this study was the suppressed anoikis resistance and migratory/invasive capacity following galectin-3 inhibition. In line with our results, Takenaka et al. reported that galectin-3 transfection into normal thyroid follicular cells potentiated anchorage-independent growth and loss of contact inhibition [30]. Additionally, treatment with low-molecular citrus pectin reduced cell migration in thyroid cancer cells [29]. Distant metastasis, either synchronous or metachronous, has a negative impact on the overall and disease-specific survival of patients with thyroid cancer [31]. In this regard, galectin-3 inhibitor therapy may be incorporated in the treatment or prevention of distant metastasis from thyroid cancer.

The phosphorylation level of ERK was shown to be downregulated following galectin-3 silencing or treatment with Td131_1 [22, 29]. However, we observed an inconsistent trend of the change in ERK phosphorylation after GB1107 and TD139 treatment. On the other hand, our data are in concordance with the results of Lin and colleagues [22] and indicate a progressive decline in AKT phosphorylation after treatment with galectin-3 inhibitors. An immunohistochemical study suggested a strong correlation between galectin-3 and *β*-catenin expression in benign and malignant thyroid tissues [32]. Consistently, we found that *β*-catenin expression was decreased in parallel to the attenuation of AKT phosphorylation. In hepatocellular carcinoma, galectin-3 overexpression enhanced metastasis through the PI3K/AKT/GSK-3*β*/*β*-catenin signaling cascade [33]. Our findings are further supported by a study demonstrating that *β*-catenin activation was highly dependent on the PI3K/AKT activity but not on the MAPK [34].

Downstream to the AKT/*β*-catenin pathway, MMP2 expression was also decreased after treatment with galectin-3 inhibitors. MMP2 is significantly overexpressed among matrix metalloproteinases in thyroid cancer and was associated with lymph node metastasis [35]. Matrix metalloproteinases contribute to the degradation of the extracellular matrix and facilitate cancer invasion. Moreover, MMP2 plays an important role in the angiogenic switch [36]. Of interest, a strong relationship between the galectin-3 network and the CD74 network was identified in a reverse-phase protein analysis [37]. We previously showed that treatment with anti-CD74 antibody inhibited cell invasion and vascular endothelial growth factor secretion in thyroid cancer cells [38]. A positive correlation between galectin-3 and CD74 expression was noted in thyroid cancer (unpublished observation). The interaction between galectin-3 and CD74 deserves further investigation in detailed molecular studies.

In conclusion, our study suggested that different galectin-3 inhibitors effectively reduce anoikis resistance and cell migration and invasion in thyroid cancer cells. Galectin-3 not only represents a diagnostic biomarker in thyroid cancer but also possesses a promising therapeutic potential for metastatic spread.

## Data Availability

The data used to support the findings of this study are included within the article or are available from the corresponding author upon request.

## References

[1] Singh Ospina N., Iñiguez-Ariza N. M., Castro M. R. (2020). Thyroid nodules: diagnostic evaluation based on thyroid cancer risk assessment. *BMJ*.

[2] De Koster E. J., De Geus-Oei L.-F., Dekkers O. M. (2018). Diagnostic utility of molecular and imaging biomarkers in cytological indeterminate thyroid nodules. *Endocrine Reviews*.

[3] Aratake Y., Umeki K., Kiyoyama K. (2002). Diagnostic utility of galectin-3 and CD26/DPPIV as preoperative diagnostic markers for thyroid nodules. *Diagnostic Cytopathology*.

[4] Lee J.-J., Wang T.-Y., Liu C.-L. (2017). Dipeptidyl peptidase IV as a prognostic marker and therapeutic target in papillary thyroid carcinoma. *The Journal of Clinical Endocrinology & Metabolism*.

[5] Thijssen V. L., Heusschen R., Caers J., Griffioen A. W. (2015). Galectin expression in cancer diagnosis and prognosis: a systematic review. *Biochimica et Biophysica Acta (BBA)-Reviews on Cancer*.

[6] Trimboli P., Virili C., Romanelli F., Crescenzi A., Giovanella L. (2017). Galectin-3 performance in histologic and cytologic assessment of thyroid nodules: a systematic review and meta-analysis. *International Journal of Molecular Sciences*.

[7] Menachem A., Bodner O., Pastor J., Raz A., Kloog Y. (2015). Inhibition of malignant thyroid carcinoma cell proliferation by Ras and galectin-3 inhibitors. *Cell Death Discov*.

[8] Blanchard H., Yu X., Collins P. M., Bum-Erdene K. (2014). Galectin-3 inhibitors: a patent review (2008-present). *Expert Opinion on Therapeutic Patents*.

[9] Xue L., Liu W., Sun Y. X. (2020). Protective effects on hypoxia reoxygenation cardiomyocytes by GLP-1R agonists via PI3K/AKT signaling pathway. *International Journal of Gerontology*.

[10] Huang T.-S., Lee J.-J., Li Y.-S., Cheng S.-P. (2019). Ethacridine induces apoptosis and differentiation in thyroid cancer cells in vitro. *Anticancer Research*.

[11] Liu C.-L., Yang P.-S., Wang T.-Y., Huang S.-Y., Kuo Y.-H., Cheng S.-P. (2019). PGC1*α* downregulation and glycolytic phenotype in thyroid cancer. *Journal of Cancer*.

[12] Cheng S. P., Lee J. J., Chang Y. C., Lin C. H., Li Y. S., Liu C. L. (2020). Overexpression of chitinase‐3‐like protein 1 is associated with structural recurrence in patients with differentiated thyroid cancer. *The Journal of Pathology*.

[13] Yang P.-S., Hsu Y.-C., Lee J.-J., Chen M.-J., Huang S.-Y., Cheng S.-P. (2018). Heme oxygenase-1 inhibitors induce cell cycle arrest and suppress tumor growth in thyroid cancer cells. *International Journal of Molecular Sciences*.

[14] Liu C.-L., Hsu Y.-C., Lee J.-J. (2020). Targeting the pentose phosphate pathway increases reactive oxygen species and induces apoptosis in thyroid cancer cells. *Molecular and Cellular Endocrinology*.

[15] Paoli P., Giannoni E., Chiarugi P. (2013). Anoikis molecular pathways and its role in cancer progression. *Biochimica et Biophysica Acta (BBA)-Molecular Cell Research*.

[16] Sciacchitano S., Lavra L., Morgante A. (2018). Galectin-3: one molecule for an alphabet of diseases, from A to Z. *International Journal of Molecular Sciences*.

[17] Colnot C., Fowlis D., Ripoche M.-A., Bouchaert I., Poirier F. (1998). Embryonic implantation in galectin 1/galectin 3 double mutant mice. *Developmental Dynamics*.

[18] Tellez-Sanz R., Garcia-Fuentes L., Vargas-Berenguel A. (2013). Human galectin-3 selective and high affinity inhibitors. Present state and future perspectives. *Current Medicinal Chemistry*.

[19] Caniglia J. L., Guda M. R., Asuthkar S., Tsung A. J., Velpula K. K. (2020). A potential role for Galectin-3 inhibitors in the treatment of COVID-19. *PeerJ*.

[20] Li J., Vasilyeva E., Wiseman S. M. (2019). Beyond immunohistochemistry and immunocytochemistry: a current perspective on galectin-3 and thyroid cancer. *Expert Review of Anticancer Therapy*.

[21] Peplau E., De Rose F., Reder S. (2020). Development of a chimeric antigen-binding fragment directed against human galectin-3 and validation as an immuno-positron emission tomography tracer for the sensitive in vivo imaging of thyroid cancer. *Thyroid*.

[22] Lin C.-I., Whang E. E., Donner D. B. (2009). Galectin-3 targeted therapy with a small molecule inhibitor activates apoptosis and enhances both chemosensitivity and radiosensitivity in papillary thyroid cancer. *Molecular Cancer Research*.

[23] Zetterberg F. R., Peterson K., Johnsson R. E. (2018). Monosaccharide derivatives with low-nanomolar lectin affinity and high selectivity based on combined fluorine-amide, phenyl-arginine, sulfur-*π*, and halogen bond interactions. *ChemMedChem*.

[24] Vuong L., Kouverianou E., Rooney C. M. (2019). An orally active galectin-3 antagonist inhibits lung adenocarcinoma growth and augments response to PD-L1 blockade. *Cancer Research*.

[25] Hsieh T. J., Lin H. Y., Tu Z. (2016). Dual thio-digalactoside-binding modes of human galectins as the structural basis for the design of potent and selective inhibitors. *Scientific Reports*.

[26] Hirani N., MacKinnon A. C., Nicol L. (2020). Target-inhibition of galectin-3 by inhaled TD139 in patients with idiopathic pulmonary fibrosis. *European Respiratory Journal*.

[27] Chalasani N., Abdelmalek M. F., Garcia-Tsao G. (2020). Effects of belapectin, an inhibitor of galectin-3, in patients with nonalcoholic steatohepatitis with cirrhosis and portal hypertension. *Gastroenterology*.

[28] Harazono Y., Kho D. H., Balan V. (2014). Galectin-3 leads to attenuation of apoptosis through Bax heterodimerization in human thyroid carcinoma cells. *Oncotarget*.

[29] Zheng J., Lu W., Wang C., Xing Y., Chen X., Ai Z. (2017). Galectin-3 induced by hypoxia promotes cell migration in thyroid cancer cells. *Oncotarget*.

[30] Takenaka Y., Inohara H., Yoshii T. (2003). Malignant transformation of thyroid follicular cells by galectin-3. *Cancer Letters*.

[31] Su D. H., Chang S. H., Chang T. C. (2015). The impact of locoregional recurrences and distant metastases on the survival of patients with papillary thyroid carcinoma. *Clinical Endocrinology*.

[32] Weinberger P. M., Adam B.-L., Gourin C. G. (2007). Association of nuclear, cytoplasmic expression of galectin-3 with *β*-catenin/wnt-pathway activation in thyroid carcinoma. *Archives of Otolaryngology-Head & Neck Surgery*.

[33] Song M., Pan Q., Yang J. (2020). Galectin-3 favours tumour metastasis via the activation of *β*-catenin signalling in hepatocellular carcinoma. *British Journal of Cancer*.

[34] Sastre-Perona A., Riesco-Eizaguirre G., Zaballos M. A., Santisteban P. (2016). ß-catenin signaling is required for RAS-driven thyroid cancer through PI3K activation. *Oncotarget*.

[35] Nakamura H., Ueno H., Yamashita K. (1999). Enhanced production and activation of progelatinase A mediated by membrane-type 1 matrix metalloproteinase in human papillary thyroid carcinomas. *Cancer Research*.

[36] Fang J., Shing Y., Wiederschain D. (2000). Matrix metalloproteinase-2 is required for the switch to the angiogenic phenotype in a tumor model. *Proceedings of the National Academy of Sciences*.

[37] Ruvolo P. P., Hu C. W., Qiu Y. (2019). LGALS3 is connected to CD74 in a previously unknown protein network that is associated with poor survival in patients with AML. *EBioMedicine*.

[38] Cheng S.-P., Liu C.-L., Chen M.-J. (2015). CD74 expression and its therapeutic potential in thyroid carcinoma. *Endocrine-Related Cancer*.

